# Emapalumab as a therapeutic intervention for Epstein–Barr virus-associated hemophagocytic lymphohistiocytosis: A case series

**DOI:** 10.1097/MD.0000000000039880

**Published:** 2024-09-27

**Authors:** Wen Wang, Xiao Yuan, Li Yu, Fuyu Pei

**Affiliations:** a Department of Pediatrics, Nanfang Hospital, Southern Medical University, Guangzhou, China.

**Keywords:** EBV infection, EBV-HLH, emapalumab, hemophagocytic lymphohistiocytosis, treatment

## Abstract

**Rationale:**

Epstein–Barr virus-associated hemophagocytic lymphohistiocytosis (EBV-HLH) is characterized by a severe cytokine storm, heightened inflammatory response, and immune-mediated damage to tissues and organs. Standard treatment protocols for hemophagocytic lymphohistiocytosis often fall short in effectively controlling EBV-HLH, leading to a need for novel therapeutic options. Emapalumab, a monoclonal antibody targeting interferon-gamma, has shown promise due to its targeted cytokine modulation capabilities and favorable safety profile. This study aimed to evaluate the efficacy and safety of emapalumab in pediatric patients with EBV-HLH.

**Patient concerns:**

The case series involved 4 pediatric patients diagnosed with EBV-HLH who did not achieve disease control despite receiving comprehensive treatment.

**Diagnoses:**

All 4 pediatric patients were diagnosed with EBV-HLH.

**Interventions:**

Emapalumab was introduced as an adjunctive therapeutic intervention alongside the HLH-94 or L-DEP regimens for these patients.

**Outcomes:**

Among the 4 patients, 1 experienced severe multiorgan dysfunction and opted to discontinue therapy. The remaining 3 patients showed controlled disease progression with significant clinical improvements following emapalumab administration. These improvements included reduced levels of inflammatory markers, normalization of blood counts and liver function, and decreased Epstein–Barr virus viral load.

**Lessons:**

The findings suggest that emapalumab may be an effective and safe treatment option for pediatric EBV-HLH. However, further research is necessary to confirm these outcomes, especially in critically ill patients.

## 1. Introduction

Hemophagocytic lymphohistiocytosis (HLH) is a clinical syndrome characterized by significantly reduced cytotoxic T lymphocytes (CTLs) and natural killer (NK) cell cytotoxicity that results in abnormal sustained activation and proliferation of CTLs and macrophages, which are ineffective in clearing viruses and other antigens.^[1,2]^ Clinical manifestations include fever, hepatosplenomegaly, lymphadenopathy, cytopenia, liver dysfunction, coagulopathy, and central nervous system involvement.^[3]^ HLH progresses rapidly and has a high mortality rate. While the pathogenesis of HLH remains incompletely understood, it is classified into primary (genetic) and secondary (acquired) types.^[4]^ Primary HLH arises from genetic defects, such as impairments in CTL and NK-cell function and mutations in genes associated with lysosomal proteins.^[5]^ Secondary HLH, on the other hand, is linked to infections (the most common trigger), tumors, and autoimmune diseases. Among patients with virus-related HLH, Epstein–Barr virus (EBV) is the most common cause.^[6]^

The specific pathogenesis of EBV-associated HLH (EBV-HLH) remains unclear, but it is generally agreed to include cytokine storms, heightened inflammatory responses, and immune damage to tissues and organs.^[7]^ Some researchers posit that EBV infection of CTLs within the body can activate a cytokine storm, leading to multisystem tissue damage.^[8]^ Currently, basic treatment of HLH involves immunosuppression and cytotoxic therapy to address the heightened inflammatory state. Recognized treatment includes HLH-1994 and HLH-2004 protocols^[9]^ as well as monoclonal antibody therapy^[10]^ and hematopoietic stem cell therapy. However, emapalumab has been reported to be a viable rescue treatment for patients with EBV-HLH.^[11]^ Additionally, Brito-Zeron et al^[12]^ suggested that anti-tumor necrosis factor therapy may be effective. However, these treatment modalities do not consistently control the progression of HLH, necessitating the exploration of new treatment strategies.

Research has indicated that interferon-gamma (IFN-γ) plays a crucial role in the development of HLH,^[13,14]^ with data from patients with primary or secondary HLH demonstrating elevated levels of IFN-γ or IFN-induced chemokines.^[15,16]^ Emapalumab, a therapeutic intervention for HLH, is a monoclonal antibody targeting IFN-γ, effectively reducing excessive inflammatory responses in patients with HLH by inhibiting the activity of IFN-γ.^[17]^ Nevertheless, existing treatment regimens have not yielded satisfactory results regarding EBV-HLH, prompting our administration of a combination of IFN-γ monoclonal antibodies in 4 patients with EBV-HLH with the aim of swiftly controlling the disease.

## 2. Case reports

### 2.1. Patient 1

Patient 1, a 6-year-old female, was admitted on August 8, 2023, with a clinical presentation of recurrent fever and trilineage hematopoietic suppression persisting for 2 weeks. Bone marrow examination prior to hospitalization revealed a significant increase in hemophagocytic activity, although flow cytometry did not detect any aberrant cellular phenotypes. Despite a week-long course of methylpednisolone administered before her admission, the patient’s condition did not improve. Upon admission, clinical examination noted mild jaundice affecting the skin and mucous membranes, as well as lymphadenopathy and hepatosplenomegaly. Hematological assessment showed profound bone marrow suppression, characterized by a white blood cell count of 0.61 × 10^9^/L, a neutrophil (NEU) count of 0.57 × 10^9^/L, hemoglobin (HGB) at 74 g/L, and a critically low platelet count of 13 × 10^9^/L. Biochemical tests indicated severe hepatic injury, with alarmingly high levels of alanine aminotransferase (ALT) at 2787 U/L, aspartate aminotransferase (AST) at 3888 U/L, direct bilirubin at 49.1 μmol/L, total bilirubin at 51.2 μmol/L, and lactate dehydrogenase at 2369 U/L. Ferritin levels (Ferr) was markedly elevated at 11901.42 ng/mL. EBV quantification returned a high viral load of 2.81 × 10^6^ copies/mL, and EBV DNA polymerase chain reaction differentiated between EBV cellular tropism as follows: T-cell (EBV-T DNA) 4.26 × 10^5^, B cell (EBV-B DNA) 1.24 × 10^5^, and natural killer cell (EBV-NK DNA) 2.61 × 10^3^, indicating an active and multifaceted EBV infection. Additionally, soluble interleukin-2 receptor (sCD25) levels were elevated at 60,639 U/mL, corroborating the diagnosis of EBV-HLH. This case typifies the aggressive and multisystem involvement of EBV-HLH, underscoring the necessity for prompt and effective therapeutic interventions.

**Figure 1.**
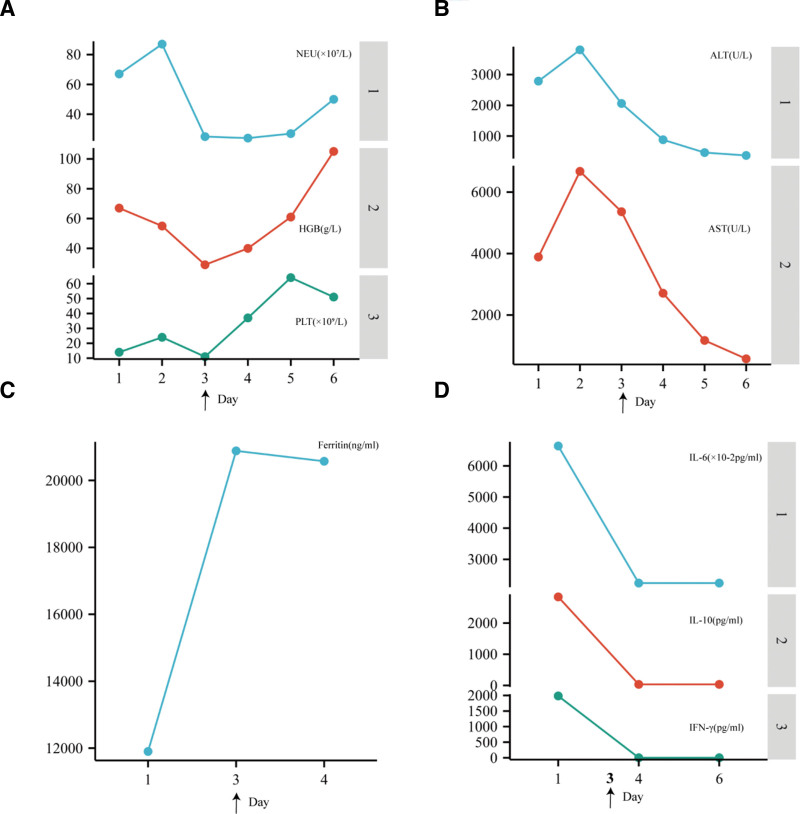
Graphical representation of important laboratory parameters and treatment in Patient 1. (A) Neutrophil count (NEU), hemoglobin (HBG), and platelet (PLT) counts. (B) Alanine aminotransferase (ALT) and aspartate aminotransferase (AST). (C) Ferritin. (D) Interleukin-6 (IL-6), interleukin-10 (IL-10), and interferon-gamma (IFN-γ). Arrows on the *x*-axis of each graph indicate the start of emapalumab treatment.

Upon admission, the patient was empirically treated with meropenem and Cancidas for anti-infective purposes. Additionally, liver protection was provided through the administration of glutathione, magnesium isoglycyrrhizinate injection, ademetionine 1,4-butanedisulfonate, L-ornithine L-aspartate granules, and vitamin C. On August 9, treatment for EBV-HLH was initiated with dexamethasone (10 mg/m^2^), ruxolitinib (2.5 mg qd), and immunoglobulin (10 g qd). Etoposide was introduced on August 10, along with plasma exchange and continuous renal replacement therapy (CRRT). Unfortunately, on August 11, the patient experienced melena 3 times and 1 episode of seizure, which could have been attributed to coagulation disorders, hepatic encephalopathy, or central nervous system involvement in HLH. At this point, the diagnosis of hemophagocytic syndrome was confirmed, along with multiple organ dysfunction and poor response to steroid therapy. To address this, emapalumab was added to the etoposide and ruxolitinib regimen, while the dose of dexamethasone was gradually reduced; plasma exchange and CRRT were continued. The dosage of emapalumab was 1 mg/kg, administered twice weekly. Following the administration of emapalumab, the patient’s hemoglobin, platelet count, and neutrophil count rebounded, and liver function improved, as shown in Figure 1. However, the patient developed epistaxis, intermittent seizures, respiratory distress, and a positive Kernig sign, necessitating tracheal intubation. During the night, the patient experienced bradycardia, progressively increasing blood pressure, and dilated pupils, indicative of intracranial hypertension, for which mannitol was administered to reduce intracranial pressure, subsequently normalizing blood pressure. Unfortunately, on August 13, the patient’s condition deteriorated with diffuse cerebral hemisphere swelling, subdural effusion in the frontal and temporal regions, and possible subarachnoid hemorrhage. As a result, the family chose to withdraw treatment due to liver failure and severe brain dysfunction, and the patient passed away.

In summary, the patient received treatment according to the HLH-1994 protocol, which included dexamethasone, VP-16, ruxolitinib, emapalumab, and immunoglobulin, along with anti-infective measures, liver protection, correction of coagulation function, and CRRT therapy. Despite the administration of multiple therapies, including emapalumab, the patient unfortunately succumbed to the disease, highlighting the need for further research on treatment strategies aimed at improving outcomes for patients with HLH.

### 2.2. Patient 2

This case report presents the clinical course of a 2-year-old male patient who was admitted to the hospital on August 22, 2023, with a persistent fever lasting 16 days. On admission, the patient exhibited jaundice, hepatosplenomegaly, coagulation disorders, elevated Ferr levels, and significantly decreased levels of white blood cells, hemoglobin, and platelets. The patient was diagnosed with EBV-HLH. Subsequent testing confirmed the diagnosis, revealing high levels of T-cell EBV DNA in peripheral blood along with positive quantitative EBV testing.

**Figure 2.**
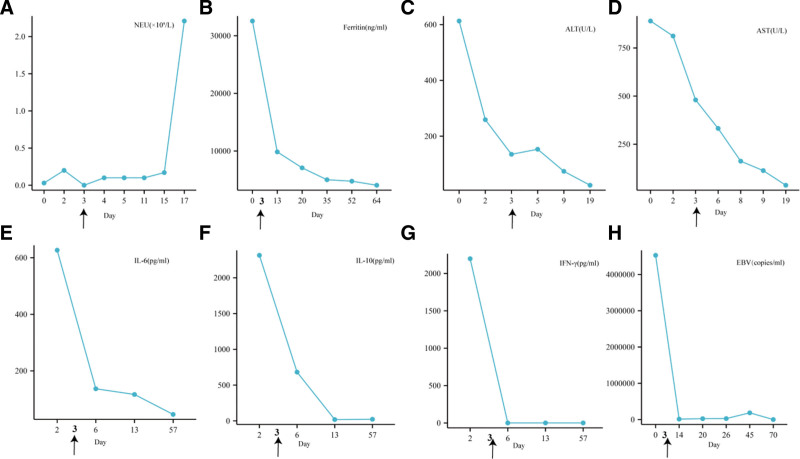
Graphical representation of important laboratory parameters and treatment in Patient 2. (A) Neutrophil count (NEU). (B) Ferritin. (C) Alanine aminotransferase (ALT). (D) Aspartate aminotransferase (AST). (E) Interleukin-6 (IL-6). (F) Interleukin-10 (IL-10). (G) Interferon-gamma (IFN-γ). (H) Epstein–Barr virus. Arrows on the *x*-axis of each graph indicate the start of emapalumab treatment.

Following the diagnosis, the patient received a comprehensive treatment approach consisting of anti-infective drugs, supportive care, liver protection, hemostasis support, and the L-DEP rescue protocol. Emapalumab administration commenced on the third day after admission. Notably, emapalumab treatment resulted in a notable decrease in inflammatory markers and Ferr levels, normalization of liver function, and resolution of all infections through supportive antimicrobial therapy. Figure 2 depicts the rapid decline in EBV levels following emapalumab treatment, highlighting its efficacy in managing EBV-HLH.

Although the patient’s condition was controlled after emapalumab treatment, complete remission was not achieved, necessitating a second cycle of L-DEP chemotherapy. Throughout the treatment, the patient’s health indicators showed varying degrees of improvement. Ultimately, the patient underwent transplant surgery on October 18 and was discharged with stable disease control, successfully returning to normal life. Despite encountering some treatment-related complications, prompt interventions effectively resolved them.

Overall, this case report underscores the effectiveness of emapalumab in the management of EBV-HLH, providing novel insights and therapeutic options for this condition.

### 2.3. Patient 3

An 8-year-old female patient presented with fever and abdominal pain accompanied by clinical manifestations such as absence of jaundice in the skin and mucous membranes, nodular rash on the lower limbs, hepatosplenomegaly, and edema. She was admitted to the hospital on October 19, 2023. Initial laboratory investigations upon admission revealed a reduced white blood cell count of 2.96 × 10^9^/L, decreased NEU count of 0.82 × 10^9^/L, low HGB of 87 g/L, and diminished platelet count of 48 × 10^9^/L. Liver function tests indicated elevated levels of ALT at 147 U/L and AST at 173 U/L, suggestive of hepatic dysfunction. Furthermore, elevated Ferr levels were indicative of inflammation and tissue damage. Quantification of EBV demonstrated increased EBV DNA levels, with abnormal peripheral blood T and B cell numbers, consistent with the diagnostic criteria for EBV-HLH.

**Figure 3.**
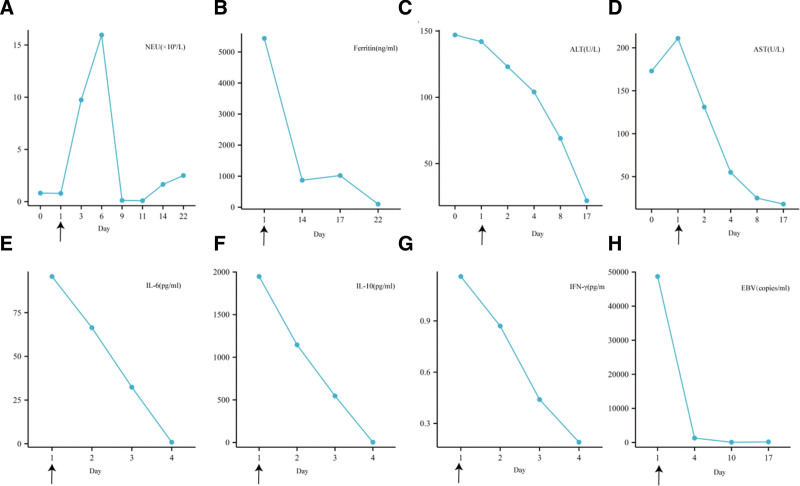
Graphical representation of important laboratory parameters and treatment in Patient 3. (A) Neutrophil count (NEU). (B) Ferritin. (C) Alanine aminotransferase (ALT). (D) Aspartate aminotransferase (AST). (E) Interleukin-6 (IL-6). (F) Interleukin-10 (IL-10). (G) Interferon-gamma (IFN-γ). (H) Epstein–Barr virus. Arrows on the *x*-axis of each graph indicate the start of emapalumab treatment.

Upon admission, the patient received medium-flow oxygen for respiratory support. Anti-infective therapy with meropenem and clarithromycin was initiated on October 20 and continued through October 26. Diuretics, including furosemide, were administered for diuresis, while dopamine and dobutamine were employed to enhance pulmonary circulation. Fresh frozen plasma was transfused to replenish coagulation factors. Expectorant therapy with ambroxol and liver protection with glutathione were provided. Chemotherapy based on the HLH-1994 protocol, comprising etoposide, dexamethasone, and ruxolitinib, commenced on October 20. The patient initially responded well to etoposide and dexamethasone treatment; however, she subsequently developed recurrent symptoms, including fever, abdominal pain, immune activation, pneumonia, and neurological manifestations. Given these challenges, emapalumab was incorporated into the treatment regimen. As depicted in Figure 3, inflammatory markers gradually declined during treatment, accompanied by the normalization of body temperature. Concurrently, improvements were observed in the patient’s blood parameters, liver function, and Ferr levels. Following emapalumab therapy, the patient’s condition was effectively managed, with resolution of inflammatory markers, normalization of body temperature, and rapid reduction in EBV levels. Subsequent follow-up confirmed the patient’s sustained health posttreatment, devoid of relapses or complications.

### 2.4. Patient 4

On October 29, 2023, a 10-year-old male patient was admitted to the hospital with intermittent fever persisting for over 20 days. Further clinical manifestations included weight loss, nodular rash on the lower limbs, and bruising on the right calf. Additionally, palpable lymphadenopathy on the left side of the neck and splenomegaly 4 cm below the costal margin were observed, accompanied by hematopoietic impairment, indicated by decreased total NEU counts, reduced HGB levels, and thrombocytopenia. Laboratory investigations revealed elevated levels of ALT, AST, and lactate dehydrogenase, indicating hepatic dysfunction, while low albumin levels and prolonged APTT, accompanied by high D-dimer levels, suggested coagulation abnormalities. Furthermore, heightened Ferr levels signified an inflammatory state.

After a comprehensive evaluation, the patient was diagnosed with hemophagocytic syndrome, prompting further relevant investigations that revealed aberrant EBV quantification, EBV subtyping, and NK-cell activity, supporting a diagnosis of EBV-HLH. The HLH-1994 protocol chemotherapy, comprising etoposide, dexamethasone, and ruxolitinib, was initiated on November 1, and emapalumab therapy was administered on the 5th day post-admission to modulate the inflammatory response and ameliorate symptoms by inhibiting IFN-γ biological activity. Concurrent interventions included anti-infective measures, correction of coagulation abnormalities, liver protection, and cardioprotective strategies.

**Figure 4.**
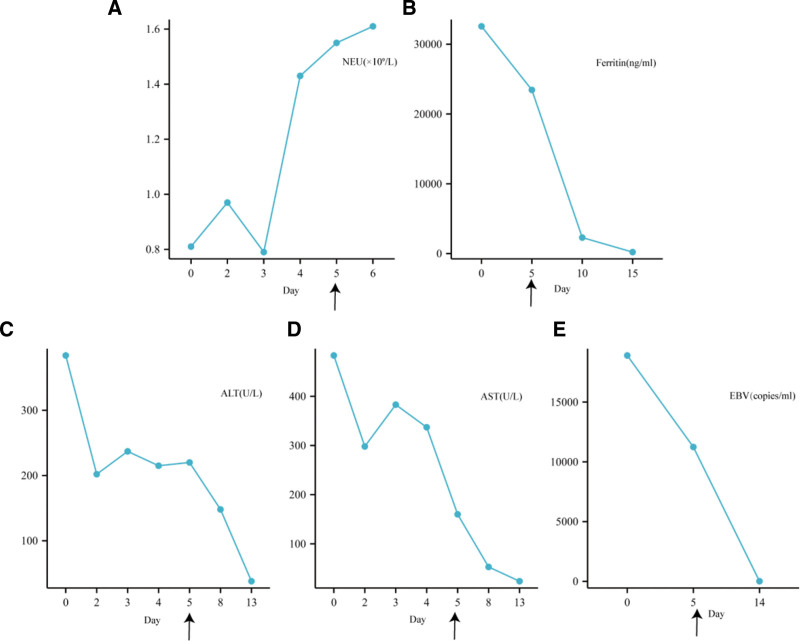
Graphical representation of important laboratory parameters and treatment in Patient 4. (A) Neutrophil count (NEU). (B) Ferritin. (C) Alanine aminotransferase (ALT). (D) Aspartate aminotransferase (AST). (E) Epstein–Barr virus. Arrows on the *x*-axis of each graph indicate the start of emapalumab treatment.

Throughout the therapeutic course, inflammatory markers, including body temperature, complete blood count, and liver function, were closely monitored. The administration of emapalumab resulted in noteworthy improvements in the patient’s condition, as evidenced by normalization of the total neutrophil count, reduction in Ferr levels, rapid decline in EBV titers, and overall disease stability, as illustrated in Figure 4. Subsequent follow-up affirmed the patient’s sustained well-being without recurrence or untoward complications, underscoring the efficacy of the treatment regimen.

## 3. Discussion

EBV-HLH is a severe immunopathological reaction triggered by an EBV infection. This condition is characterized by systemic inflammation and dysfunction of multiple organs, with pathological features, such as extensive proliferation of activated lymphocytes and histiocytes capable of phagocytosing tissues in locations such as the bone marrow, lymph nodes, and spleen.^[18]^ This leads to severe systemic inflammation and dysfunction of multiple organs. The exact pathogenesis of EBV-HLH remains incompletely understood, but it is currently believed to be linked to aberrant T-cell and NK-cell functions following EBV infection.^[19]^ These cells fail to efficiently clear the virus, resulting in excessive production of cytokines like IFN-γ, TNF-α, and IL-6, which trigger systemic inflammation and a cytokine storm. Research conducted by Cohen^[20]^ revealed that EBV infection of B lymphocytes induces polyclonal proliferation of cytotoxic T lymphocytes, subsequently activating macrophages and the immune system to secrete large quantities of cytokines. Studies by Kasahara et al^[21]^ have shown that EBV infection of CD8+ T lymphocytes and NK cells leads to the expression of CD21 antibodies, rapidly triggering the release of cytokines from various tissue cells. Simultaneously, the activated lymphocytes and phagocytes engulf blood cells in significant numbers, resulting in clinical manifestations of anemia, decreased platelets, and coagulation abnormalities.

Diagnosis of EBV-HLH necessitates fulfillment of 2 criteria. First, the diagnostic guidelines for HLH must be satisfied. Second, confirmation of EBV infection is imperative.^[22]^ In all cases examined in this study, the clinical presentations of the pediatric patients unequivocally met the diagnostic criteria for HLH, and elevated EBV viral load coupled with early antigen IgG positivity provided compelling evidence of EBV infection, culminating in the definitive diagnosis of EBV-HLH. Currently, the primary therapeutic approach for EBV-HLH adheres to the induction therapy outlined in HLH-1994 and HLH-2004 treatment regimens to quell excessive inflammatory responses. Noteworthy findings indicate that early administration of etoposide chemotherapy significantly diminishes the mortality rate associated with EBV-HLH.^[23]^ For instance, in the case of Patient 1, initial anti-infective therapy comprised meropenem and caspofungin, alongside liver-protective agents like glutathione, magnesium isoglycyrrhizinate, ademetionine, L-ornithine-L-aspartate granules, and vitamin C. These interventions aimed to impede viral replication, ameliorate liver impairment, and enhance liver function. Subsequent immunomodulatory therapy involved the use of dexamethasone, ruxolitinib, and immunoglobulin. Dexamethasone, a potent anti-inflammatory agent, suppresses immune cell activation, thereby mitigating inflammation. Ruxolitinib, a JAK1/2 inhibitor, impedes cytokine signaling, attenuating cytokine storms.^[24]^ Immunoglobulin confers immune protection and reduces the risk of infection. Etoposide chemotherapy was subsequently administered to curb the activation and proliferation of lymphocytes and phagocytes. Plasma exchange and CRRT were also employed to eliminate viruses and cytokines from the bloodstream, ameliorating multi-organ dysfunction. Despite the comprehensive therapeutic interventions implemented, challenges emerged as the patient developed hematochezia and seizures during treatment. Given the potential for coagulation abnormalities, hepatic encephalopathy, and central nervous system impairment in HLH, these complications could not be discounted. These occurrences underscore the inadequacy of the treatment strategies employed to effectively manage the patient’s condition. The severity of the viral infection, exacerbated immune response triggering cytokine storms, and ensuing multi-organ dysfunction likely contributed to therapeutic inefficacy. Considering the escalating clinical complexity, a decision was made to escalate the administration of emapalumab. Emapalumab, a monoclonal antibody that targets IFN-γ, directly inhibits the interferon’s biological activity, thereby mitigating cytokine storms.^[25]^ EBV-HLH presents a grave malady, necessitating prompt and accurate diagnosis coupled with proactive and holistic intervention strategies. Despite the exhaustive therapeutic modalities employed, encompassing anti-infective measures, liver protection, immunomodulation, chemotherapy, plasma exchange, CRRT, and emapalumab, the patient’s condition persisted uncontrolled, culminating in severe encephalopathy and fatality. This poignant clinical scenario underscores the imperative for further exploration of optimal treatment timing and strategies for EBV-HLH patients, underscoring the criticality of promptly addressing hemophagocytic manifestations in such individuals.

In Patients 2, 3, and 4, we implemented a comprehensive therapeutic approach encompassing anti-infective agents, intravenous immunoglobulin support, and hemostatic therapy. The primary aim of these treatment regimens was to eradicate the sources of infection, correct coagulation abnormalities, and provide supportive care. Furthermore, the administration of furosemide, with its diuretic properties, contributed to the enhancement of pulmonary circulation. Fresh frozen plasma was administered to restore coagulation factors and mitigate the risk of bleeding and associated complications. Ambroxol and glutathione were employed to facilitate expectoration and provide hepatic protection. Regarding the HLH chemotherapy regimen, patients were treated according to either the HLH-1994 or HLH-2004 protocols, incorporating etoposide, dexamethasone, and ruxolitinib. Etoposide, functioning as a purine analog, exerts inhibitory effects on cell proliferation and DNA synthesis, thereby suppressing aberrant cellular growth.^[26]^ Dexamethasone, a glucocorticoid agent, exhibits anti-inflammatory and immunosuppressive properties, aiding in the modulation of excessive immune activation.^[27]^ Ruxolitinib, an effective targeted therapeutic agent, impedes viral replication and cellular proliferation.^[28]^ After an inadequate initial treatment response, the patients received emapalumab therapy, a monoclonal antibody targeting IFN-γ. Prior to this intervention, the patients presented with persistent fever, dysregulated immune activation, and markedly elevated levels of inflammatory markers and Ferr. posttreatment, the patients demonstrated reductions in body temperature, gradual normalization of inflammatory markers, decreased Ferr levels, and restoration of hepatic function. These outcomes underscore the favorable impact of emapalumab in the therapeutic regimen. By obstructing the IFN-γ signaling pathway, emapalumab hampers abnormal T-cell activation and cytokine release, thereby alleviating systemic inflammatory responses and tissue damage. Clinical evidence supports the substantial amelioration of symptoms, disease progression, and enhanced survival rates in EBV-HLH patients following emapalumab treatment.^[10]^ Nevertheless, some patients developed infections during emapalumab administration aimed at IFN-γ blockade, necessitating resolution through supportive antimicrobial interventions as well as discontinuation of etoposide and dexamethasone. This underscores that while emapalumab therapy yields efficacy, it also entails specific therapeutic risks and complexities.

The therapeutic approach to EBV-HLH holds paramount significance due to its etiological nature. Given that EBV-HLH is precipitated by EBV infection, the use of antiviral medications is imperative for treatment. Acyclovir and ganciclovir have demonstrated efficacy against herpesviruses and have shown favorable outcomes in the management of infectious mononucleosis caused by EBV.^[29]^ However, the use of acyclovir in antiviral therapy for EBV-HLH remains contentious.^[30]^ Notably, in this study, no significant improvement in the EBV viral load was observed following antiviral treatment. Recent investigations have highlighted the potential utility of monoclonal antibodies for targeted therapy in refractory EBV-HLH patients exhibiting persistent elevation of EBV viral load after undergoing chemotherapy.^[31]^ In a specific case, the patient tested negative for EBV DNA after receiving targeted therapy with emapalumab monoclonal antibody, corroborating the research findings. This phenomenon may be attributed to the drug’s suppression of the cytokine storm, consequently inhibiting the replication and dissemination of the EBV virus via the host’s immune system, thereby further mitigating the severity of the disease. Furthermore, the early administration of methylprednisolone and intravenous immunoglobulin therapy has been observed to create opportunities for subsequent induction therapy. This effect might be associated with the diminished activity of helper T lymphocytes, the promotion of regulatory T lymphocyte activation, and the reduced secretion of inflammatory cytokines. Additionally, the blockade of tissue cell Fc receptors may further attenuate phagocytic activity. These observations are consistent with the treatment regimen recommended in HLH-2004, which involves “high-dose methylprednisolone combined with intravenous immunoglobulin,” suggesting a potential role in this context. Given the life-threatening nature of EBV-HLH, which poses a significant risk to children, the presence of genetic abnormalities may necessitate the consideration of hematopoietic stem cell transplantation for refractory recurrent patients exhibiting poor responses to conventional chemotherapy.

EBV-HLH is a rapidly progressing and often fatal disease. Clinical challenges often lead to misdiagnosis, resulting in delays in treatment initiation. Early and accurate diagnosis, along with timely implementation of treatment protocols, is essential for enhancing patient outcomes and prognoses. This study presented clinical observations of 4 pediatric patients diagnosed with EBV-HLH who did not achieve disease control despite receiving comprehensive treatment. Subsequently, the anti-IFN-γ monoclonal antibody emapalumab was introduced as an adjunctive therapeutic intervention. Patient 1, who was diagnosed with hemophagocytic syndrome along with multiple organ dysfunction and showed a poor response to steroid therapy, still did not experience disease control even after the addition of emapalumab, ultimately withdrawal of treatment. However, in Patients 2, 3, and 4, who presented with persistent fever, dysregulated immune activation, and markedly elevated levels of inflammatory markers and Ferr, the disease was effectively controlled with the use of emapalumab. The findings revealed a reduction in inflammatory markers, recovery of blood counts and liver function, and a decrease in EBV levels following emapalumab administration, indicating its potential efficacy. Nevertheless, Patient 1 encountered severe multi-organ dysfunction during treatment, and care was discontinued, highlighting a potential limitation in the therapeutic response of emapalumab for critically ill patients. In all cases, we did not observe significant emapalumab-related side effects, indicating a favorable safety profile for the treatment. Overall, the study outcomes suggest that emapalumab may have a positive effect on reducing inflammatory markers, controlling disease progression, and improving clinical symptoms in pediatric patients with EBV-HLH. However, the variability in patient responses, including 1 case of severe adverse outcomes, underscores the need for further research to optimize treatment strategies and identify patient subgroups that may benefit most from emapalumab therapy. These findings also highlight the potential of combining emapalumab with other treatment modalities, such as chemotherapy or hematopoietic stem cell transplantation, to enhance patient outcomes.

This study has several limitations that should be considered when interpreting the findings. Firstly, the small sample size of 4 pediatric patients limits the generalizability of the results to a broader population with EBV-HLH. The lack of a control group further restricts the ability to draw definitive conclusions about the efficacy and safety of emapalumab in comparison to other treatment modalities. Additionally, the variability in the patients’ clinical presentations and prior treatments may also introduce confounding factors that affect the outcomes. Moreover, the use of emapalumab poses certain challenges and unresolved issues that necessitate further investigation to establish its therapeutic benefits and safety profile. Future studies with larger cohorts, controlled designs, and longer follow-up periods are necessary to validate these findings and better understand the role of emapalumab in managing EBV-HLH.

In summary, emapalumab as an adjunctive therapy for EBV-HLH demonstrates promising efficacy and safety profiles. However, given the limited sample size and scarcity of severely ill patients, additional validation is essential to substantiate our conclusions. Subsequent studies should aim to enlarge the sample size, delve deeper into the therapeutic potential of emapalumab in EBV-HLH treatment, and explore alternative treatment modalities to enhance patient outcomes and survival rates.

## Author contributions

**Conceptualization:** Fuyu Pei.

**Data curation:** Wen Wang, Xiao Yuan.

**Formal analysis:** Wen Wang, Li Yu.

**Writing – original draft:** Wen Wang, Xiao Yuan, Li Yu.

**Writing – review & editing:** Wen Wang, Fuyu Pei.
